# Quality indicators for radiation oncology

**DOI:** 10.1111/1754-9485.13373

**Published:** 2022-03-03

**Authors:** Susan V Harden, Kim‐Lin Chiew, Jeremy Millar, Shalini K Vinod

**Affiliations:** ^1^ Peter MacCallum Cancer Centre Melbourne Victoria Australia; ^2^ School of Public Health and Preventive Medicine Monash University Melbourne Victoria Australia; ^3^ Macarthur Cancer Therapy Centre Campbelltown Hospital Campbelltown New South Wales Australia; ^4^ South Western Sydney Clinical School UNSW Ingham Institute for Applied Medical Research Liverpool New South Wales Australia; ^5^ Radiation Oncology Alfred Health Melbourne Victoria Australia; ^6^ Cancer Therapy Centre Liverpool Hospital Liverpool New South Wales Australia

**Keywords:** benchmarking, clinical key performance indicators, quality indicators, quality measures, radiation oncology, radiotherapy

## Abstract

Quality Indicators, based on clinical practice guidelines, have been used in medicine and within oncology to measure quality of care for over twenty years. However, radiation oncology quality indicators are sparse. This article describes the background to the development of current national and international, general and tumour site‐specific radiation oncology quality indicators in use. We explore challenges and opportunities to expand their routine prospective collection and feedback to help drive improvements in the quality of care received by people undergoing radiation therapy.

## Introduction

The delivery of high‐quality evidence‐based cancer care is integral to achieving optimal outcomes for patients. National clinical practice guidelines (CPGs) for cancer care and optimal cancer care pathways are designed to assist clinical decision‐making and guide best practice. However, adherence to guidelines and time taken to implement practice‐changing trials as standard of care can vary across both individual practitioners and healthcare organisations (HCOs). This information can only be apprehended if what actually happens in the real world compared to best practice guidelines can be measured and reported.

Quality Indicators (QIs) in health care have been developed to measure compliance with defined evidence‐based quality standards across many medical specialties including oncology. QIs in cancer care are used to help understand the quality of care being provided, identify areas for improvement and measure change. Almost all QIs in cancer care are developed around the Donabedian healthcare quality domains of structure, processes and outcomes first published over 40 years ago.^1^ Structural QIs measure the quality of the setting in which care is provided including workforce and equipment; process QIs measure how care is actually delivered along the patient pathway compared to guideline recommended care, including diagnosis, treatment assessment, planning, delivery and follow‐up; and outcome QIs measure how the care that was provided affects the patient’s health status.

The American Institute of Medicine (IOM) ‘Crossing the quality chasm’ report on quality of health care identified six components to high‐quality health care delivery: with care needing to be effective (evidence‐based), efficient, safe, timely, equitable and patient‐centred.^2^

The ultimate goal of QIs is to measure and identify gaps in quality of care in order to facilitate quality improvement with increasing compliance to QIs.

## Oncology QIs

**Fig. 1:**
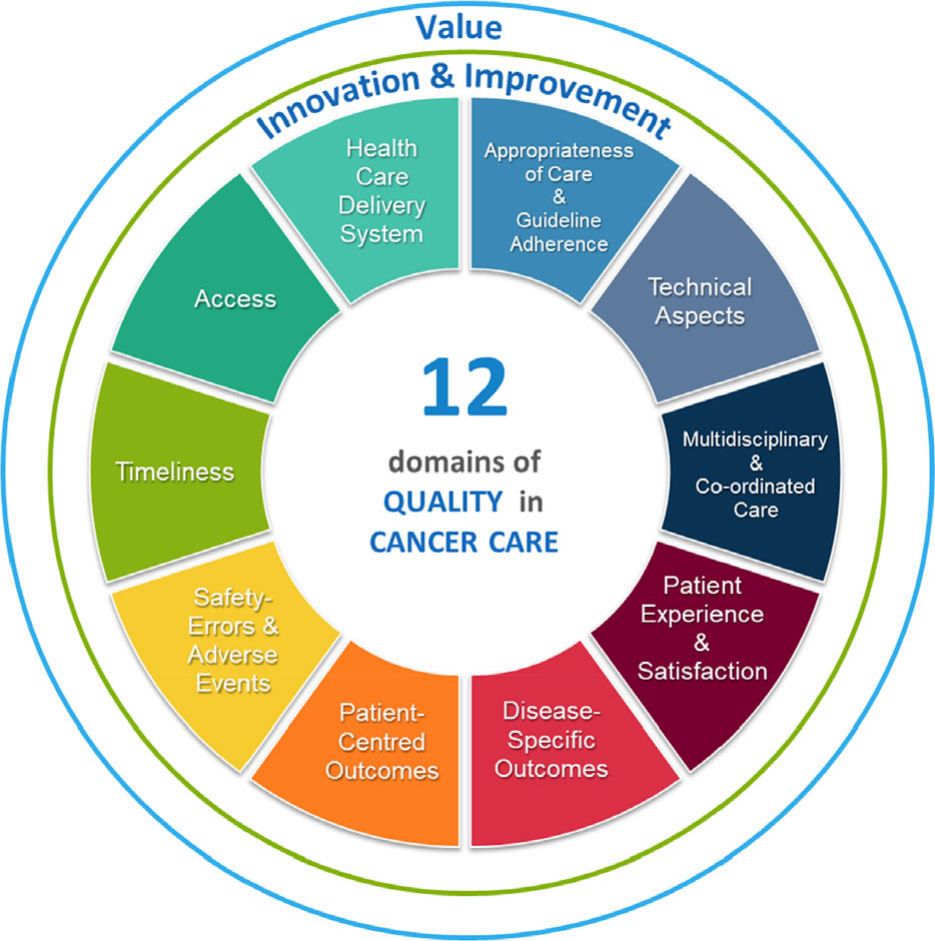
Conceptual framework for the quality of cancer care. Reproduced with permission.

The frameworks for QIs have been applied in the cancer care setting (Fig. 1). To date, the majority of oncology QIs are process‐based and disproportionately weighted towards the effectiveness and safety of surgical procedures with very limited radiation oncology QIs (ROQIs).^3^, ^4^, ^5^, ^6^ Albert and Das highlighted the particular challenges for developing and updating relevant oncology QIs to incorporate all the evolving changes in best practice with evidence‐based trials of novel treatments and technologies.^4^ At the time of their report, QIs developed for measuring quality of care in oncology tended to be tumour site‐specific, focussing along the whole patient pathway, from diagnosis, through treatment to survivorship and end‐of‐life care, for example, as based on standards as developed for lung cancer by an international consortium.^7^ Tumour specific oncology QIs are also reported in retrospective annual patterns of care audits by a number of countries.^8^, ^9^, ^10^, ^11^

An updated framework for modern cancer care was recently proposed by Chiew *et al*. to incorporate 12 areas for quality of care expanding upon the Donabedian model of structural (timeliness, access and healthcare delivery systems) process (appropriateness of treatment, technical, multidisciplinary discussion/coordination of care, patient reported experiences (PRE) and satisfaction) and outcome domains (disease‐specific survival, patient reported outcomes (PRO), safety and adverse events, with all areas also needing to include value and innovation/improvement.^12^

Oncology QIs are commonly developed and derived via an established methodology of evidence‐based literature search, modified Delphi process and expert consensus/concordance voting. In addition to being evidence‐based, Oncology QIs must be feasible to collect, have validity with clinician ‘buy‐in’ and potentially be able to show variation over space and time. The Australian Council on Healthcare Standards (ACHS) has recently published a consensus for collecting cancer care clinical QIs from administrative data sets excluding ROQIs.^13^

## Radiation oncology quality indicators (ROQIs)

Radiation therapy (RT) is a technical specialty and has a long history of quality; each radiation oncology department has quality standards and quality assurance processes to ensure patient safety particularly with emerging technologies and novel treatment delivery systems used to deliver state of the art high‐quality care. Many of these aspects are covered in other papers in this issue, all links within the ‘quality’ chain for RT delivery. Measuring the breadth of quality of care in RT relies upon a spectrum of measures that inevitably overlap from machine quality assurance, radiation oncology (RO) peer review, departmental quality standards through to population level indicators of quality.

The following discussion will touch upon ROQIs that can be used for self‐assessment, quality improvement and accreditation within departments but in particular will focus on population metric ROQIs, which can be reliably and feasibly collected at state or commonwealth level for benchmarking practice of RO departments against other equivalent national and international HCOs. Population level ROQIs can be particularly useful for measuring quality of care in terms of equitable access to novel technologies, implementation of practice‐changing trials and assessing value to the state and patient population.^12^ For example, hypofractionation for intermediate risk prostate cancer adds value for the state/commonwealth and for the patient, but HCOs themselves receive less reimbursement for delivering reduced fractionation. However, in value terms, the money saved by hypofractionation could potentially be redirected back to HCOs to fund high‐cost equipment such as MR‐linacs.

It is very important to classify ROQIs by their level of ‘coverage’ and intended use. The scope for ROQIs can range from the level of performance for a linac or practice for a RO consultant, through HCO accreditation up to patterns of care and comparisons of RO delivery across the population. ROQIs at these different levels will have different aims and, from guiding physics and engineering at one end, and public health policy, funding and standards at population national level. ROQIs and the data elements collected at each extreme are obviously different, but in the middle there are important distinctions. For example, measuring rates of use of single fraction palliative RT for bone metastases nationally is *different* to monitoring use of single fractions within HCOs. As a population metric ROQIs are general ‘should’ statements, not ‘must’ statements; it is not likely that the clinical circumstances of the patient can be integrated for a population ROQI whereas as an institutional metric, the age of patient, primary, gender, systemic control of disease, previous treatment, disease‐free interval, proximity to cord influencing decision‐making could be obtained. Nevertheless, at a population level an index of proportion of single fraction over institutions may be revealing: differences between HCOs are unlikely to be related to huge differences in patient population features in centres, but more likely reflect differences in practice.^14^, ^15^

## General ROQIs

In USA, Hayman et al defined and distinguished ROQIs from their related radiation oncology quality measures (ROQMs), based on prior Patterns of Care studies, which evolved into the Quality Research in Radiation Oncology (Q‐RRO) programme.^16^ Initially focussing on several tumour sites, commonly treated with definitive curative intent RT, the Q‐RRO programme is now used HCO level for internal self‐assessment and accreditation and also used at population level for national benchmarking. They acknowledged that ROQIs designed for this higher level of reporting inevitably lack the internal level of details for absolute confirmation of quality. They described a core set of evidence‐based outcome‐linked process measures for cancers ‘cured’ by RT as primary treatment (cervix, breast, lung and prostate cancers), for emerging technologies at that time (high dose rate (HDR) brachytherapy, intensity modulated RT (IMRT) and a feedback process for ROQIs. They emphasised the need for prospective and frequent measurement, and desirable characteristics such as importance, scientific acceptability, reliability, validity, useability and feasibility. The ROQIs were further subdivided into general or tumour specific and by the intended quality target (individual RO/patients/HCO/national).

Albert and Das updated and reviewed the development for ROQIs to develop consensus QIs especially for rapidly emerging complex technologies to ensure their safe delivery.^17^ They performed an extensive literature, guideline and website search and summarised the relevant published ROQIs (Table 1 and 2). They also highlighted some difficulties for collecting ROQIs at a population level, including the lack of standards for reporting diversity and complexity of RT, and also suggested possible solutions^18^

**Table 1:** General radiation oncology quality indicators

RT Pathway	ROQI	Domain	References
Pre‐treatment Clinical	MDM discussion	Structure, process Multidisciplinary care coordination	ACHS^26^, SEOR^23^, van Lent^20^, Cionini^22^
	Staging/minimum medical record data available	Structure, process, appropriateness of care	ACHS^26^, SEOR^23^, Cionini^22^, Albert^17^, Gabriele^21^
	Treatment based on clinical practice guidelines/published data	Process, appropriateness of care	Albert^17^
RT HCO provider organisational aspects	Treatments/RT sessions per linac WORKLOAD Equipment to deliver IMRT IGRT, Treatments per RO	Structure, healthcare delivery system	SEOR^23^, UK RTDS^29^, vanLent^20^, Cionini^22^, Gabriele^21^
	Linac time lost for unscheduled interruptions/rescheduling of RT/planned but patient didn’t start	Structure, healthcare delivery system	SEOR^23^, van Lent^20^, Cioini^22^, Gabriele^21^
RT pre‐planning and planning	Waiting time to start treatment/access RT/from simulation to first fraction	Structure, timeliness	ACHS^26^, SEOR^23^, vanLent^20^, Cionini^22^, Gabriele^21^
	% referred to another centre due to lack of suitable resource	Structure, access	SEOR^23^
	Signed consent (and documentation of risks) AND RT INTENT	Outcome, Patient‐centred	SEOR^23^, Allbert^17^
	Peer review of contouring and dosimetry	Process, Technical, Safety	ACHS^26^, Albert^17^
	Physics QC and dosimetry checks and equipment QA especially for IMRT/VMAT/IGRT	Structure, process, Technical, Safety	vanLent^20^, Cionini^22^, Gabriele^21^
	Patient screened for pain prior /acute symptoms during RT?	Process, outcome, Patient‐centred	Albert^17^
RT delivery	Motion management (gating, 4DCT)	Structure, process, Technical	ACHS^26^
	Single fraction for bone metastasis (<10) or justification why not single fract or >10	Value, Patient‐centred	ACHS^26^, Choosing wisely^27^, UK RTDS^29^, Albert^17^
	RT or surgery within 24 hours of diagnosed cord compression	Process, Patient‐centred	Albert^17^
	Avoid WBRT if SRS too; avoid toxic local RT if also distant mets	Process, Patient‐centred	Choosing wisely^27^
	Treatment delay/prolongation	Process, Timeliness	ACHS^26^, SEOR^23^
	Use of special techniques (IMRT, SBRT, SRS, TBI, under GA, Intraoperative RT, adaptive RT FOR PLANNING AND DELIVERY	Structure, process, innovation, Technical	SEOR^23^, UK RTDS^29^, van Lent^20^, Cionini^22^, Albert^17^
	Use of verification on set (IGRT) CBCT	Process, Technical	SEOR^23^, Gabriele^21^
	% retreatment or re‐irradiation	Process, Safety, Technical	SEOR^23^
Post‐Treatment	Communication of RT summary sent to treating team	Process, Multidisciplinary care coordination	Albert^17^
	>grade 3 CTCAE chronic complication	Outcome, Safety, Patient centred	SEOR^23^
	Patient satisfaction	Outcome, Patient experience	SEOR^23^, vanLent^20^, Cionini^22^
	RT HCO RO publications and impact	Outcome, Innovation	SEOR^23^, vanLent^20^
	Patients entering trials	Outcome, innovation	SEOR^23^, van Lent^20^, Gabriele^21^
	Overall Survival (with reference to RT HCO volume)	Outcome, Disease‐specific outcomes	Tchelebi^39^

**Table 2:** Tumour site‐specific radiation quality indicators

Tumour site	RT Pathway	ROQI	Quality Domain	References
PROSTATE	Pre‐treatment and Clinical	Documentation of pre‐treatment PSA	Process, appropriateness of care	Tsiamis^25^, Albert^17^
		Documentation of clinical stage, TNM and Gleason primary and secondary/tertiary grade	Process, appropriateness of care	Tsiamis^25^, Albert^17^
		Documentation of risk‐specific staging investigations for high risk prostate cancer	Process, appropriateness of care	Tsiamis^25^, Albert^17^
		Different treatment options discussed with patient for localised including active surveillance for low‐risk disease?	Process	Albert^17^ UK NPCA^10^, Choosing Wisely^27^
	Treatment	Men with high risk disease receiving local active treatment	Process	Tsiamis^25^
		Men undergoing conventionally fractionated should receive at least 74 Gy to the prostate	Process, appropriateness of care	Tsiamis^25^, SEOR^23^, Q‐RRO^36^
		Men undergoing radical RT should receive IMRT/VMAT	Process, technical, safety, patient‐centred	Tsiamis^25^, Albert^17^
		Men receiving EBRT should be treated on high energy lincac>6MV, with DVH calculations for EBRT and post‐implant dosimetry for BT	Process, technical	Q‐RRO^36^, Albert^17^
		Men undergoing EBRT should have daily IGRT (fiducial markers or CBCT)	Process, technical, patient‐centred	Tsiamis^25^, Q‐RRO^36^
		Men with intermediate risk disease offered hypofractionation	Process, patient‐centred	UK NPCA^10^, UK RTDS^29^, PCOR‐ANZ^11^
		Men with high risk disease offered RT to pelvic nodes	Process	UK NPCA^10^
		Men with high risk disease should not get LDR brachytherapy	Process, appropriateness of care	Tsiamis^25^
		Men receiving LDR should get over 140/145 Gy Iodine 125	Process, appropriateness of care	Tsiamis^25^, SEOR^23^
		Men with low‐risk disease receiving EBRT should not get ADT	Process, appropriateness of care	Tsiamis^25^
		Men with high risk disease should have long course ADT >2 years	Process, appropriateness of care	Tsiamis^25^, ACHS^26^, Q‐RRO^36^, Albert^17^
	Salvage	Post‐RP, men without M1 disease should be offered salvage RT	Process, appropriateness of care	Tsiamis^25^
	Post‐treatment	Document PSA within 1 year post‐RT	Outcome	Tsiamis^25^
		Patient seen in clinic for follow‐up assessment within 1 year	Outcome	Tsiamis^25^
		Assessment of PRO and QoL at 1 year	Outcome, Patient‐centred	Tsiamis^25^, UK NPCA^10^
		Lower GI admissions for toxicity (up to 2 years post‐RT)	Outcome, patient‐centred	NPCA^10^, ^32^
BREAST	Pre‐treatment	Multiple multidisciplinary aspects of care for diagnosis and initial treatment	Process, Structure	Best^24^
		Receipt of adjuvant RT after surgery (when no SACT) within 12 weeks	Process, timeliness	Best^24^
		RT to LN as well as breast/chest wall when N+	Process, appropriateness of care	Best^24^
		Delivery of boost to primary when age<50 or when positive margin	Process, appropriateness of care	Best^24^
		Node negative cases receiving adj RT to whole breast after BCS	Process, appropriateness of care	Best^24^
		Use of heart dose constraints, heart DVH, access to DIBH, plans with max point dose‐limited to 110%	Process, Technical	Best^24^
	Treatment	Guidelines for complex cases including LN fractionation, implants, wound healing. Peer review of these and internal mammary inclusion	Structure	Best^24^
		Boost to resection cavity 16 Gy/8# or 10 Gy/4‐5#	Process, appropriateness of care	Best^24^
		Use of hypofractionation for adjuvant RT after conservative surgery	Process, value, patient‐centred	Best^24^, SEOR^23^, UK RTDS^29^, Choosing wisely^27^
		Receipt of adjuvant RT within 1 year of conservative surgery	Process, Appropriateness of care	Albert^17^
	Post‐Treatment	Hormone therapy use for stage Ic‐IIIC ER and PR positive cases	Process, Appropriateness of care	Albert^17^
		Complete follow‐up documented following RT after breast conservations (including mammography, healthcare provider responsible for surveillance, survivorship plan and referral back to GP	Process, multidisciplinary	Albert^17^, Best^24^
LUNG		Use of CTPET and brain imaging prior stage III curative intent	Process	UK NLCA^9^, Q‐RRO Komaki^35^
		Use of SABR for stage I and II NSCLC	Process, Value, patient‐centred	SEOR^23^, UK NLCA^9^, ^30^
		Use of concurrent chemoRT NSCLC	Process, Appropriateness of care	UK NLCA^9^, ^31^
		Use of doses over 60 Gy for conventional RT NSCLC	Process, Appropriateness of care	Q‐RRO Komaki^35^
		Use of twice daily RT for L‐SCLC and PCI	Process, Appropriateness of care	Q‐RRO Komaki^35^
		Define at least 2 OAR	Process	Albert^17^
RECTAL		Patients with locally advanced disease receiving RT within 6 months of diagnosis/ presurgery	Process, Appropriateness of care	Albert^17^
PANCREAS		Use of chemo RT when no surgery and define at least 2 OAR	Process, Appropriateness of care	Albert^17^
Head and Neck		People treated with IMRT	Structure, Technical	SEOR^23^
CERVIX		Use of chemoRT for curative intent treatments	Process, Appropriateness of care	Albert^17^

The Canadian Partnership for Quality Radiotherapy set out a useful decision tree model for identifying ROQIs adding ‘Other’ to the Donabedian domains of structure, process, outcomes.^19^ This provided a logical structure for identifying and collating valid ROQIs, and also defined how the ROQI pertains to a specific ‘target’ namely patient/staff/equipment or HCO. Their paper focuses on describing a clear process for developing ROQIs rather than listing them. A European group performed a literature search with stakeholder feedback to evaluate the feasibility of collecting 33 ROQIs for international benchmarking of RT HCOs. Their pilot feasibility study at 4 RT HCOs found that 14/33 ROQIs were robust in terms of clarity, availability and discriminative ability.^20^

ROQIs have also been developed and subsequently updated in Italy where they were selected and modified by an expert working group rather than a Delphi process.^21^, ^22^ Their proposed ROQIs (Table 1) covered two structural QIs (IMRT delivering linacs, workload relative to workforce), ten process QIs (Multidisciplinary meeting (MDM) discussion, multimodality imaging, clinic documentation, QA, dosimetric controls for IMRT, image‐guided RT (IGRT), adaptive RT) and two outcome QIs (proportion treated in trials and machine uptime). These were then validated in four Italian radiation centres. Their ROQIs did not include items for measuring toxicity or patient reported experience (PRE) or outcome (PRO) measures.

Perhaps the most recent and relevant ROQI development paper comes from the Spanish Society of Radiation Oncology (SEOR).^23^ They carried out a systematic literature search and 2‐round Delphi process for 28 ROQIs. 26 gained a consensus from the expert group as best measuring quality for RO and being feasible for the majority of Spanish HCOs information systems. These appear relevant for modern international RO and HCO comparisons with 8 structure, 15 process and 6 outcome ROQIs (Table 1 and 2). Importantly, the ROQIs cover both general and tumour specific ROQIs, as well as including ROQIs for brachytherapy, re‐irradiation, PROs and clinical trials participation. However, the proposed ROQIs have yet to be reported in general use, which is a required step to confirm feasibility in the real world to allow reporting to HCOs in a timely manner.

## Tumour specific ROQIs

Several tumour site‐specific sets of ROQIs have been proposed. These are commonly developed subsequent to the evident paucity of ROQIs within general tumour site‐specific oncology QI publications.^3^, ^5^, ^17^

A Canadian group looked at Breast ROQIs initially with a literature search, followed by a modified Delphi process and then national survey.^24^ For 22 Tier 1 indicators – over 33% voted them as important and for 11 Tier 2 votes were lower but peer reviewed and measurable). 20/33 QIs were specifically about the RT decision‐making and planning and treatment pathway (Table 2).

Prostate ROQIs for the Australia and New Zealand Prostate Cancer Outcomes Registry (PCOR‐ANZ) were similarly developed via systematic literature review and Delphi process with 17 ROQIs endorsed.^25^ PCOR‐ANZ already provides twice‐yearly QI feedback to clinicians and surgical units in Australia and New Zealand and plan to commence feedback to reports to RT HCOs by the end of 2021. Although the initial range of potential indicators included those pertaining to structure and outcome as well as process, in fact, the final endorsed list did not contain measures for structure, and were all regarding process, except for one outcome measure.

## Existing ROQIs in use

Although there are a number of publications describing the development of consensus sets of ROQIs, there are very few publications on their actual use and their impact on improving quality of care. It can be challenging to measure general or tumour site‐specific ROQIs from automated reports. In depth detailed audit of individual case notes may be required to ascertain quality.

In fact Australia has reported on a small set of radiation oncology clinical indicators for over 20 years including indicators assessing quality of the consultation process, treatment process and delivery.^26^ However, these are very broad in their remit and do not cover technical aspects relating to quality of care. Furthermore, contribution is voluntary and out of over 100 RT providers in Australia, just 8 HCOs participated, so measuring impact on quality of care is limited.

RANZCR has published a set of five standards for ‘Choosing wisely’ in radiation oncology.^27^ These include consideration of hypofractionation for adjuvant RT after breast conserving surgery (BCS) for breast cancer, discussion of active surveillance for low‐risk prostate cancer, avoiding extended fractionation for treating bone metastases, avoiding use of whole brain RT adjuvant to stereotactic radiosurgery (SRS), and avoiding extensive locoregional therapy when there is metastatic disease and lack of local symptoms. A recent study by Ong et al looked at 3 of the 5 choosing wisely measures that were feasible to evaluate using the administrative Victorian RT minimum data set (VRMDS). They showed that over time, since publication, the use of breast hypofractionation has increased, use of adjuvant whole brain RT after SRS decreased as did the use of more than 10 fractions for treating bone metastases.^28^

Internationally, high level population ROQIs are mandated, collected and routinely reported in the UK through automated treatment machine data submitted to the national radiotherapy data set (RTDS) for the NHS Quality Innovation Productivity and Prevention (QIPP) process. The emphasis is on value based health care within an overstretched National Health Service (NHS) and accordingly QIPPs include use of hypofractionation for breast and prostate cancer and use of single fractions to treat bone metastases.^29^ These QIPPs are published as quarterly real‐time dashboards on the NHS CancerStats website and summarised on their public CancerData website.

Linkage of the UK RTDS to the UK national cancer registry has also enabled tumour‐specific reporting of population ROQIs linked to national audits for prostate and lung cancer.^9^, ^10^ This data linkage has allowed the lung cancer audit to publish on underuse of concurrent chemoradiation for stage III NSCLC and inequality of access to SABR for early stage lung cancer.^30^, ^31^ Most recently the prostate audit combined PRO and administrative data for lower GI procedures two years after prostate RT.^32^

In the USA, the Veteran Affairs Radiation Oncology Quality Surveillance programme based on the Q‐RRO methodology has successfully collected and reported retrospectively on data to measure quality and patterns of care for prostate and lung cancer collected from hospital electronic medical records in addition to radiation treatment planning and management systems.^33^, ^34^ Patterns of care have also been reported directly by Q‐RRO and retrospective analysis of the National Cancer Database.^35^, ^36^, ^37^

## Gaps in ROQIs

Historically tumour specific sets of oncology QIs contain limited ROQIs. In addition to possible under‐recognition of the importance of RO in cancer care and underutilisation,^38^ this may also be due in part to the fact RO systems and data storage are separate to general hospital medical records and perceived as not easily accessible.

Many ROQIs focus on process but, even so, ensuring high quality of care is delivered when there is increasing complexity in planning and delivery with the introduction of new technologies is difficult at population level and may still require internal HCO ROPS and detailed audit to confirm quality. The simple fact that IMRT or IGRT was used (automated data items routinely submitted to state health departments) does not necessarily guarantee the quality of that IMRT or IGRT. Likewise, yes/no documentation of peer review for the outlining of volumes or MDM discussion and staging does not necessarily imply quality.

Gaps in ROQIs relating to structure and outcome are a particular challenge. Increasing the number of meaningful ROQIs relating to structure also requires collection to be feasible and reproducible. One possible example, as suggested by SEOR is to consider a ROQI relating to RT facility volume for highly complex definitive curative intent RT, like for surgery.^23^ A retrospective multivariate analysis from the American national cancer database recently showed that for certain tumour sites treated with definitive RT (Lung and head and neck cancers) this correlated with improved overall survival.^39^ Therefore, should the number of cases planned per year by individual ROs or by RT provider, be included in internal standards and accreditation?

As general hospital medical records become increasingly electronic, the feasibility of linking clinical information pre‐treatment (staging and MDM discussion) and patient outcomes post‐treatment (including late toxicity data, PROs and survival) with the RT linac data (including total prescribed dose and fractionation) may be improved.

However, this may still be aspirational and in the recent PCOR‐ANZ ROQI Delphi process, a number of ROQIs felt to be important, were discarded because they were not thought feasible on a standardised large‐scale at present.^25^ The three important but discarded measures were MDM documentation, pre‐treatment patient quality of life assessment and patient satisfaction with treatment choice.

## Addressing Gaps in ROQIs: Hurdles and Barriers to routine ROQI collection

With regard to state or commonwealth level reporting of ROQIs, there are a number of barriers and hurdles, some more easy to address than others.

The first is to establish what is currently collectible from administrative RT data sets. For example, two of the five RANZCR Choosing Wisely indicators were not easily evaluable by an administrative state RT data set.^28^ Linkage of the state minimum RT data set to the corresponding state cancer outcome registry for PCOR‐ANZ would have enabled one more indicator to be assessed. Particularly in Australia, a further barrier to using linkage of RT data sets to administrative registry data is time lag from collection to use for the latter.

When ROQIs are used for benchmarking, there is a need for data harmonisation. For example, in Victoria VRMDS data fields include prescription dose but there is no record of prescribing method. A median dose to PTV is different to prescribing to a 60 or a 90% isodose line. The same dose prescribed, but to these different points, is very different. Such lack of harmonisation means inter‐comparisons are difficult, and even more so with benchmarking across states or internationally. It is important to ensure that there is not a ‘tower of Babel’ problem across multiple jurisdictions reporting the ‘same’ ROQI differently. Even within a single state, in the example of Victoria, there are differing standards for reporting ‘time to treatment’ for both targets and metrics. So, proportions based on days or weeks over target wait times may need to be reported to different authorities, making comparisons or discriminating between care difficult if not impossible. This is problematic with both feasibility of measurement and benchmarking, since more than one QI measuring the same thing needs to be calculated.

This definition harmonisation difficulty is a problem when considering structural indicators, which might be valid for comparisons and benchmarking across jurisdictions and across time. For example, one might consider an indicator capturing the availability of ‘image‐guidance’. Does this mean the use of developed‐film portal images, or does it mean the use of video or surface marker respiratory gating and breath‐hold techniques? And does it mean these are always used in suitable cases (and how are these defined) or just on the ones, which might be treated on specific machines (i.e. maybe only half of suitable cases?).

It is also very difficult to develop population level ROQIs for brachytherapy, molecular RT or to collect PRE and PRO measures from people who have been treated with RT. As discussed previously, assessing non‐technical ROQIs regarding coordination of care and MDM discussion and safety‐related outcome measures is difficult without linkage to hospital electronic medical records. Linkage of separate state administrative data sets data at population level may also require specific patient consent in Australia.

It will also be challenging to standardise a consistent collection of population ROQIs across all states and territories to allow commonwealth level reporting, in particular for international benchmarking. In Australia the ACHS has offered ROQIs for many years, however, in their most recent report, just eight organisations submitted data as ‘voluntary’ participants. There has to be ‘buy‐in’ for any ROQIs and local quality improvement initiatives to drive and implement change. This may be best developed through RANZCR in collaboration with the Australian Institute of Health and Welfare (AIHW) who have already successfully worked with states and territories to pilot the collection and now routine annual reporting of radiotherapy waiting times achieving full coverage of public providers and high coverage of private providers.

The rapidly evolving nature of best practice in radiation oncology with emerging complex technologies and updated evidence from new trials is also a challenge for ensuring ongoing relevance and validity of ROQIs. This may be best developed through RANZCR, perhaps by extending and regularly updating the current PRAT tool to include evolving evidence‐based tumour site‐specific ROQIs, although this may be better suited to measure institutional quality of tumour site ROQIs rather than population ROQIs. State minimum radiotherapy data sets could also be regularly reviewed and updated to incorporate novel data items for emerging technology such as MR‐linac adaptive RT and proton beam therapy.

Finally, actually reporting ROQIs back to departments and HCOs is a challenge in itself: it needs to be done in a prospective, frequent and easily visualisable manner. Individualised dashboards with clear presentation of data together with comparison of local ROQIs to those from the highest performing centres can be a very helpful tool for implementing change and measuring improvement.^40^

## Recommendations/Conclusion

For evidence‐based population level ROQIs to be used in routine practice in Australia, they need to be prospectively collected and regularly reported, harmonised across all states and territories, ideally with linkage to data from respective state cancer registries. RT providers and HCOs will need to be motivated to collect and submit non‐mandated ROQIs. Endorsement by national stakeholder organisations and possible incorporation into the national accreditation process is worthy of consideration. The potential reward is to be able to measure that increased compliance with ROQIs leads to improved outcomes and higher quality of care.

## Data Availability

Data sharing is not applicable to this article as no new data were created or analyzed in this study.
